# Effects of exercise combined with brain stimulation on hand function in children with cerebral palsy: a meta-analysis of randomized controlled trials

**DOI:** 10.7717/peerj.20670

**Published:** 2026-01-29

**Authors:** Shuoqi Li, Shenhao Guo, Ruihan Wang, Jiayuan Ma, Hu Lou

**Affiliations:** 1 School of Sports Science, Nantong University, Nantong, China; 2 Department of Physical Education, Hebei University of Architecture, Zhangjiakou, China

**Keywords:** Brain stimulation, Hand function, Cerebral palsy, Children

## Abstract

**Background:**

Cerebral palsy (CP) is a paediatric condition generally characterized by persistent motor disabilities in hand function. This review examined the impact of exercise with and without brain stimulation on hand function in children with CP.

**Methodology:**

A systematic literature search was conducted from January 2010 to June 2025 across four electronic databases: Web of Science, Scopus, PubMed, and EBSCO. This review established the inclusion criteria as follows: 1. Children with CP; 2. Randomised controlled trial; 3. Exercise with and without brain stimulation; 4. Measurements included gross motor function (GMF), fine manual control (FMC) and grip strength (GS) evaluated at pre- and post-intervention. The quality of the included studies was assessed using the Cochrane Risk of Bias tool. For data analysis, the standardized mean difference (SMD) was selected as the appropriate effect size index, and RevMan 5.4 software was employed to analyze the mean differences in the data extracted from the included articles. (Registration number: CRD420251106181).

**Results:**

The results showed that exercise with brain stimulation comprising more than 16 sessions could notably improve GS (SMD, 1.38 (0.88, 1.88), *p* < 0.05, I^2^ = 0%), whereas that comprising fewer than 10 sessions did not demonstrate a statistically significant effect (SMD, 0.19 (−0.29, 0.67), *p* = 0.44, I^2^ = 0%). Consequently, brain stimulation intervention could substantially enhance FMC (SMD, 0.46 (0.15, 0.76), *p* < 0.05, I^2^ = 47%). Subgroup analysis also presented that exercise with transcranial direct current stimulation (tDCS) resulted in a significant improvement in FMC (SMD, 0.71 (0.29, 1.14), *p* < 0.05, I^2^ = 49%) compared to exercise with repetitive transcranial magnetic stimulation (SMD, 0.19 (−0.25, 0.63), *p* = 0.09, I^2^ = 47%).

**Conclusion:**

This review demonstrated that exercise with brain stimulation could significantly enhance hand function in children with CP. Specifically, more than 16 sessions has greater benefits for GS, and the tDCS may confer benefits for FMC.

## Introduction

Cerebral palsy (CP) is a paediatric condition generally denoted by enduring motor disabilities. Children with CP may exhibit neuromuscular issues, which are contingent upon the type and severity of the condition. Examples of these issues include impaired coordination, contractures, muscle weakness, and spasms. Consequently, daily activity and motor function-related deficits can occur due to these issues (Ostensjø, Carlberg & Vøllestad, 2004). This observation indicates that hand function constitutes a critical component of motor function, encompassing several activities. Examples of these activities are reaching targets, manipulating, grasping, and releasing objects. Although children with CP must integrate their visual perception skills and movement sequences to execute these tasks efficiently, several challenges are constraining the upper limb function in these children. These challenges include a limited range of motion in the joints, muscle tone changes, and joint contractures (Hanna et al., 2003). The identified constraints may then augment the dependence of children with CP in their everyday activities by hindering play and self-care activities (Figueiredo et al., 2020).

Hand function rehabilitation is usually employed to foster independence, avert additional deformities, enhance the overall quality of life, and improve manual capabilities among individuals (Burgess et al., 2021). This rehabilitation can also enhance upper limb function within children with CP, encompassing numerous brain stimulation and exercise therapies. Notably, exercise therapy is a widely utilised treatment approach among clinical practitioners and researchers, underscoring the integration of affected upper limb and task-related exercises (Ilieva & Ilieva, 2020). Multiple studies have reported that children with CP can significantly benefit from this type of therapy, potentially facilitating motor function and neural remodelling. Two frequently employed strategies of brain stimulation therapy are transcranial direct current stimulation (tDCS) and repetitive transcranial magnetic stimulation (rTMS). These techniques are non-invasive methods for brain stimulation that deliver low-intensity stimulation to targeted brain regions *via* magnetic poles or electrodes positioned on the scalp. The tDCS enhances neuronal membrane potential through weak DC polarization, resulting in anodic enhancement and cathodic inhibition of cortical excitability; The rTMS induces induced currents through pulsed magnetic fields, which bidirectionally regulate cortical activity according to frequency, both of which work by regulating neural plasticity (Chiara et al., 2019). Hence, neural network activity and neurotransmitter release in specific brain regions are positively impacted (Brunoni et al., 2022). Furthermore, repetitive brain stimulation can lead to enduring alterations in cortical, potentially improving neurological and musculoskeletal disorder-related impairments (Hilderley et al., 2023; Zhong et al., 2025).

Currently, the correlation between exercise-brain stimulation (exercise combined with brain stimulation) and hand function in children with CP remains ambiguous. Certain studies have suggested that exercise-brain stimulation enhances fine manual control (FMC) and grip strength (GS) in children with CP compared to brain stimulation alone (Salazar Fajardo et al., 2022; Ebrahimabadi, Ghaderian & Askary Kachoosangy, 2024). Nonetheless, no notable difference between the two groups has been recorded in several studies (Wu et al., 2022; Gupta et al., 2023). At present, there is no meta-analysis to specifically study the synergistic effect of combined exercise and brain stimulation on different components of hand function in children with CP. Thus, this review examined the impact of exercise (in the presence and absence of brain stimulation) on hand function in children with CP using meta-analysis.

## Survey Methodology

### Data sources and study selection

**Figure 1:**
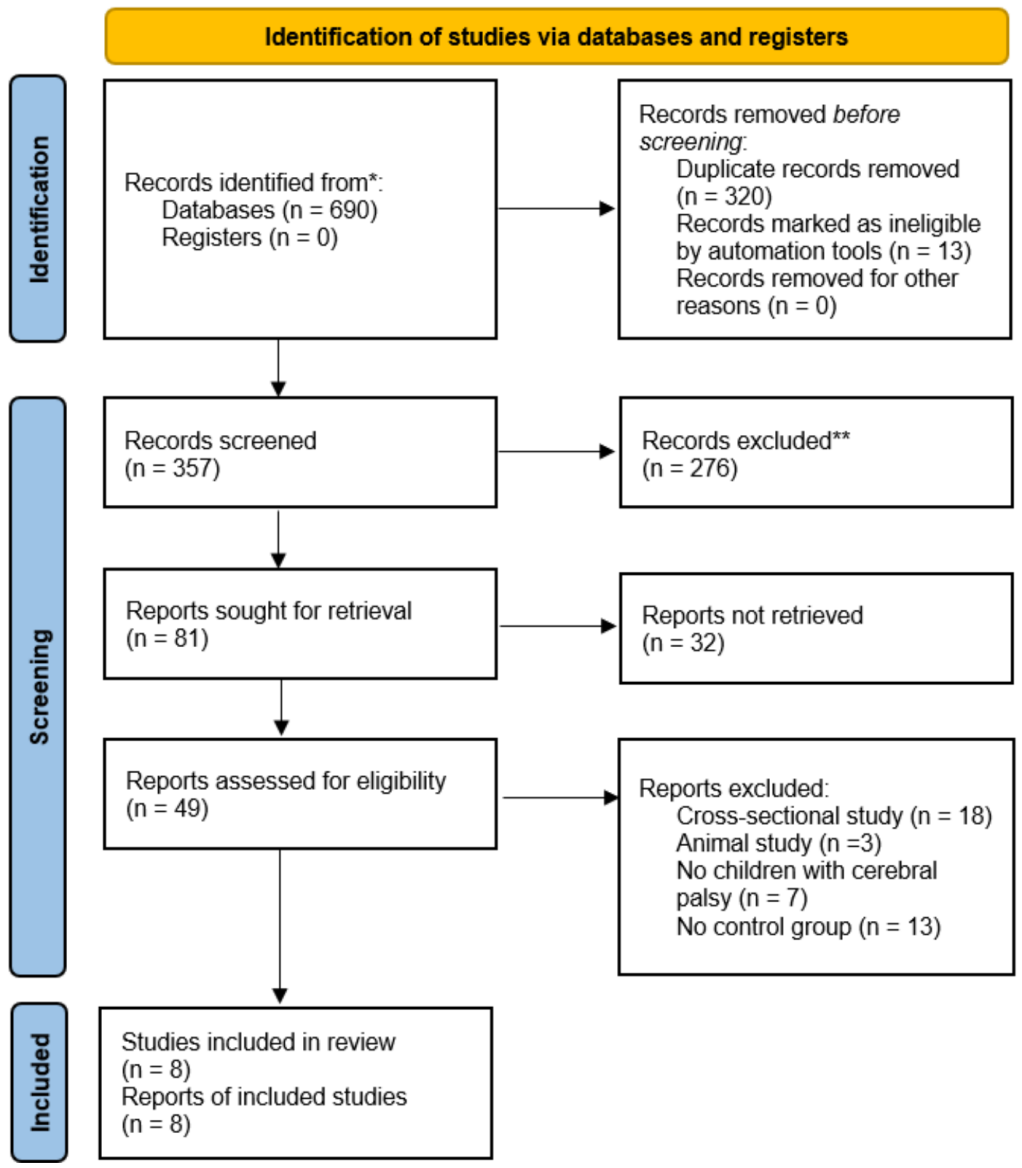
Flow diagram of the search results using the Preferred Reporting Items for Systematic Reviews and Meta-Analysis (PRISMA).

A systematic literature search was performed between January 2010 and June 2025 across four electronic databases: (i) Web of Science, (ii) Scopus, (iii) PubMed, and (iv) EBSCO. The most recent search update occurred on 29th June 2025, utilising two keywords: (i) “Cerebral palsy” and (ii) “Stimulate”. Information specialists initially developed the search strategies, with outcomes and comprehensive protocols and outcomes recorded (Appendix A). Subsequently, two independent researchers (S.L. and S.G.) conducted an initial screening of abstracts and titles obtained from the databases. The full-text articles were then assessed according to established inclusion and exclusion criteria. These same reviewers conducted quality assessment and data extraction. In instances of disagreement among reviewers, a third investigator (H.L.) was consulted to achieve consensus. The reference lists were then manually examined for further relevant articles upon confirming the included publications. This review protocol was also recorded in the international Prospective Register of Systematic Reviews (PROSPERO) (CRD420251106181). Figure 1 presents the process of study selection and preparation following the Preferred Reporting Items for Systematic Reviews and Meta-Analysis (PRISMA) requirements.

### Inclusion and exclusion criteria

This review established the inclusion criteria as follows:

(i) Randomised controlled trials.
(ii) Children with CP.
(iii) Articles comparing exercise-brain stimulation as the experimental (EXP) group and exercise with sham-brain stimulation as a control (CON) group.
(iv) Measurements included gross motor function (GMF), GS, and FMC evaluated at pre- and post-intervention.
(v) Test indicators were documented as mean (standard deviation) and/or mean (95% confidence interval (CI)).
(vi) Full-text articles were documented in English language.

In contrast, the exclusion criteria involved excluding conference proceedings, abstracts, and poster presentations from this review.

### Quality assessment

The methodological quality of the included articles was investigated through the Cochrane risk of bias assessment tool (Higgins et al., 2019). Several processes could be accomplished by employing this tool, including examining blind outcome, generating random sequences, introducing additional biases, handling incomplete outcome data, concealing allocation, selective reporting, and participant with personnel blinding (Li et al., 2023). Items were then evaluated as “yes”, “no”, or “unclear” to assess the quality of the articles.

### Data extraction

Each article contained relevant information, which was extracted and organised into a table (see Table 1). The gathered variables involved brain stimulation protocol, exercise protocol, duration, age, gender, and equipment.

**Table 1:** Characteristics of included studies.

Study	Age (y)	Gender	Duration	Exercise program	Brain stimulation program	Equipment	Index
Ebrahimabadi, Ghaderian & Askary Kachoosangy (2024)	EXP: 7.6 ± 3.2 CON: 6.8 ± 2.5	28M/22F	4 weeks; 5x/week	Occupational therapy exercises, hand function exercises such as stretching and grasping, 45 min	EXP: tDCS, 1.5 mA, Anode O1 area, cathode O2 area, 20 min CON: Sham tDCS, 20 min	tDCS (Active Tek, USA)	GMF, GS, FMC
Salazar Fajardo et al. (2022)	EXP: 4.9 ± 2.3 CON: 5.8 ± 1.7	EXP: 5M/9F CON: 7M/3F	5 weeks 3x/week	Neurodevelopmental treatment, 30 min	EXP: tDCS, 1 mA, Anode M1 region, cathode contralateral orbital region, 20 min CON: Sham tDCS, 20min	tDCS (Ybrain Inc., Korea)	GMF, FMC
Fan et al. (2024)	EXP: 4.6 ± 0.7 CON: 4.7 ± 0.5	EXP: 9M/7F CON: 13M/3F	12 weeks; 5x/week	Action observation training, 6 body movements, 30 min	EXP: rTMS, 1 Hz, 30 min unaffected M1 area, 80% RMT CON: Sham rTMS, 30min	NA	GMF
Gillick et al. (2018)	EXP: 13.2 ± 3.7 CON: 12.3 ± 4.5	EXP: 5M/5F CON: 4M/6F	10 days	CIMT, shaping functionality and developing motor skills, 120 min	EXP: tDCS, 0.7 mA, Anode SO area, cathode M1 area, 20 min CON: Sham tDCS, 20min	tDCS (Soterix Medical, USA)	GS, FMC
Gupta et al. (2023)	EXP: 8.7 ± 2.8 CON: 8.6 ± 3.2	EXP: 13M/10F CON: 17M/6F	4 weeks 2x/week	CIMT, shaping functionality and developing motor skills, 120 min	EXP: rTMS, 1 Hz, 20 mins unaffected M1 area, 90% RMT CON: Sham rTMS, 20min	rTMS (Magventure, Denmark)	GS, FMC
Hassan et al. (2024)	EXP: 11.0 ± 2.0 CON: 12.2 ± 2.1	EXP: 6M/8F CON: 3M/11F	4 weeks; 4x/week	WBVT, Stand barefoot, with both feet parallel and bent 30 degrees. 18 Hz, amplitude of 12 mm, 20 mins	EXP: tDCS 2 mA, Anode M1 region, cathode contralateral orbital region,20 mins CON: Sham tDCS, 20min	NA	GS
He et al. (2024)	EXP: 6.5 ± 2.4 CXP:7.4 ± 2.9	EXP: 12M/3F CON: 11M/5F	4 weeks; 5x/week	Routine rehabilitation treatment, 30 min	EXP: rTMS, 1 Hz, 20 mins, unaffected M1 area, 90% RMT CON: Sham rTMS, 20min	rTMS (Youde Medical Company, China)	GMF
Wu et al. (2022)	EXP: 4.2 ± 0.9 CON: 3.7 ± 1.05	EXP: 6M/11F CON: 8M/10F	10 days	CIMT, Tug of war, shooting competition, balloon passing, 180 min	EXP: rTMS, 1 Hz, unaffected M1 area, 20 mins, 90% RMT CON: Sham rTMS, 20 mins	rTMS (Yiruide Company, China)	FMC

**Notes.**

EXP experimental group CON Control group M Male F Female CIMT Constraint-induced movement therapy tDCS Transcranial direct current stimulation rTMS Repetitive transcranial magnetic stimulation GMF Gross motor function GS Grip strength FMC Fine manual control

### Data analysis

Data analyses were conducted utilising RevMan 5.4 Cochrane Collaboration (2020). Likewise, mean difference (MD) or standardised mean difference (SMD) were applied for analysis involving continuous variables. In instances where various measurement tools or units were employed for the similar outcome, the aggregated statistic chose the SMD with a 95% CI. Otherwise, the MD with a 95% CI was computed in instances where identical measurement tools or units were utilised across articles. An inverse method for data processing was also used in cases of continuous data exhibiting opposite directions of effect (higher or lower scores indicated improved outcomes in certain articles). This approach was achieved by reversing the mean and standard deviation prior to the recombination of results.

The I^2^ statistic was implemented for evaluating the heterogeneity among study results, in which high and low heterogeneity thresholds were concluded for the I^2^ statistic values of 50%, respectively (Li et al., 2023). Particularly, a random effects model was utilised if I^2^ > 50%. This outcome implied that the sources of heterogeneity should be examined using a sensitivity analysis. Nevertheless, heterogeneity among the articles was deemed acceptable if I^2^ ≤ 50% (Li et al., 2023). The meta-analysis also established a significance level of *p* < 0.05. In studies that provided the standard error, mean, and sample size, the standard error was converted to standard deviation following data extraction. Subsequently, the subgroups of GS and FMC were analyzed according to the intervention duration and brain stimulation type. The authors of the articles were also contacted through email to acquire the missing data. Only the reported data were assessed in cases in the absence of missing data.

## Results

### Eligibility of studies

This review collected 690 articles from Web of Science, Scopus, PubMed, and EBSCO databases. The articles were then imported into Mendeley Desktop software (version 1.19.8), resulting in 357 articles after the removal of duplicates. A preliminary screening of titles and abstracts also identified 49 articles, with 41 articles excluded following a full-text review. Exclusions were then applied for four primary reasons: (i) seven articles did not include children with CP, (ii) 13 articles lacked a CON group, (iii) 18 articles were cross-sectional studies, and (iv) three articles were for animals. Consequently, the final analysis comprised eight articles. Figure 1 presents the comprehensive literature selection flowchart. All selected articles obtained ethical approval from their respective institutions for their protocols. Two investigators (S.L. and S.G.) also conducted the calibration activity to verify the consistency of their assessments, in which the Cohen kappa coefficient (*k*) calculated among the two investigators was 0.917. Overall, the final eight articles comprised 147 male and 119 female participants, with 134 and 132 children in the EXP and CON groups, respectively. This review also determined that four articles applied tDCS and rTMS, respectively. Meanwhile, the minimum and maximum durations were 10 days and 12 weeks, respectively.

### Quality assessment

**Figure 2:**
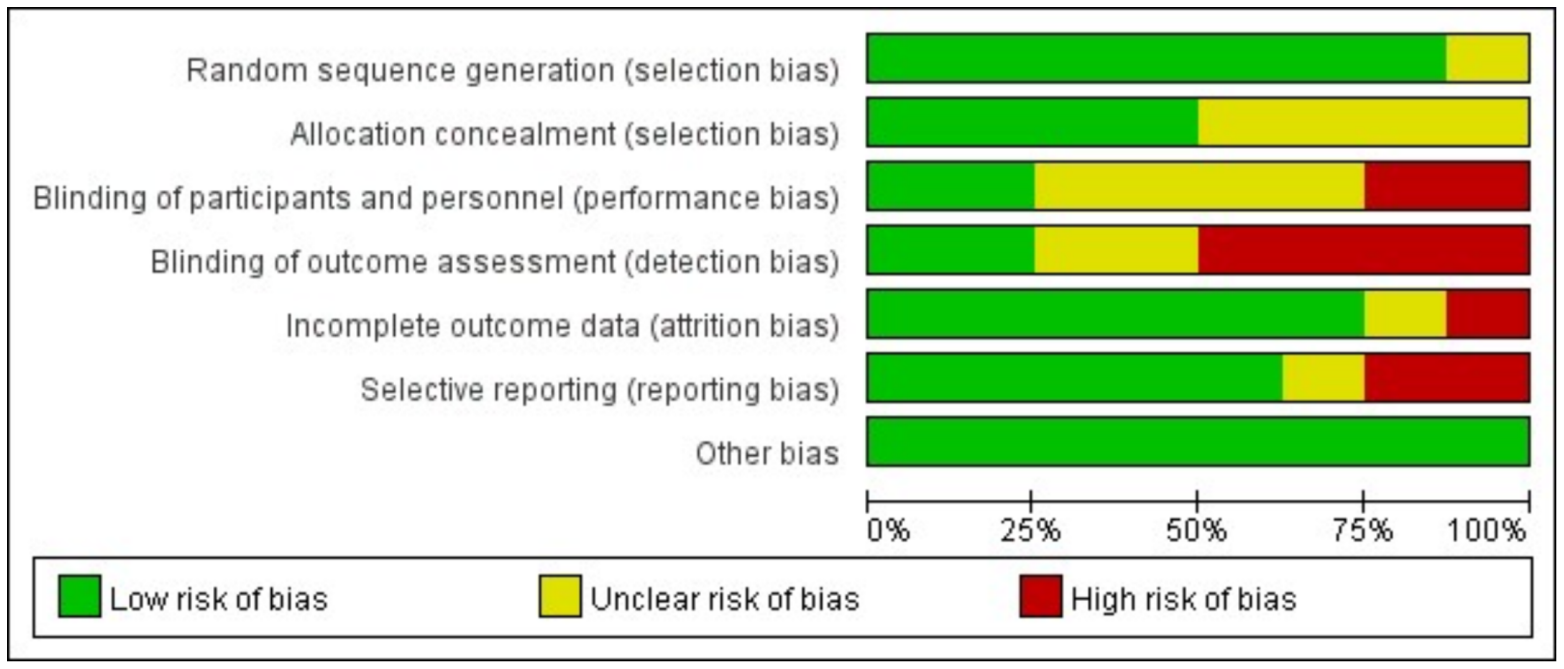
Analysis of risk of bias according to Cochrane Collaboration guideline.

Figure 2 displays an evaluation of the methodological quality and potential bias risk of the selected articles, in which the overall quality was comparatively high. The proportions of low, high, and unclear risk of bias were also 60.72%, 16.07%, and 23.21%, respectively. Moreover, this figure illustrated the risk of bias associated with each article chosen in the analysis.

### Sensitivity analysis

Sensitivity analysis was performed by excluding studies one by one and modifying the type of analysis model. The sensitivity analysis then indicated no significant changes in the outcomes across the groups in the meta-analysis, confirming the reliability of the findings.

### Quantitative synthesis

**Figure 3:**
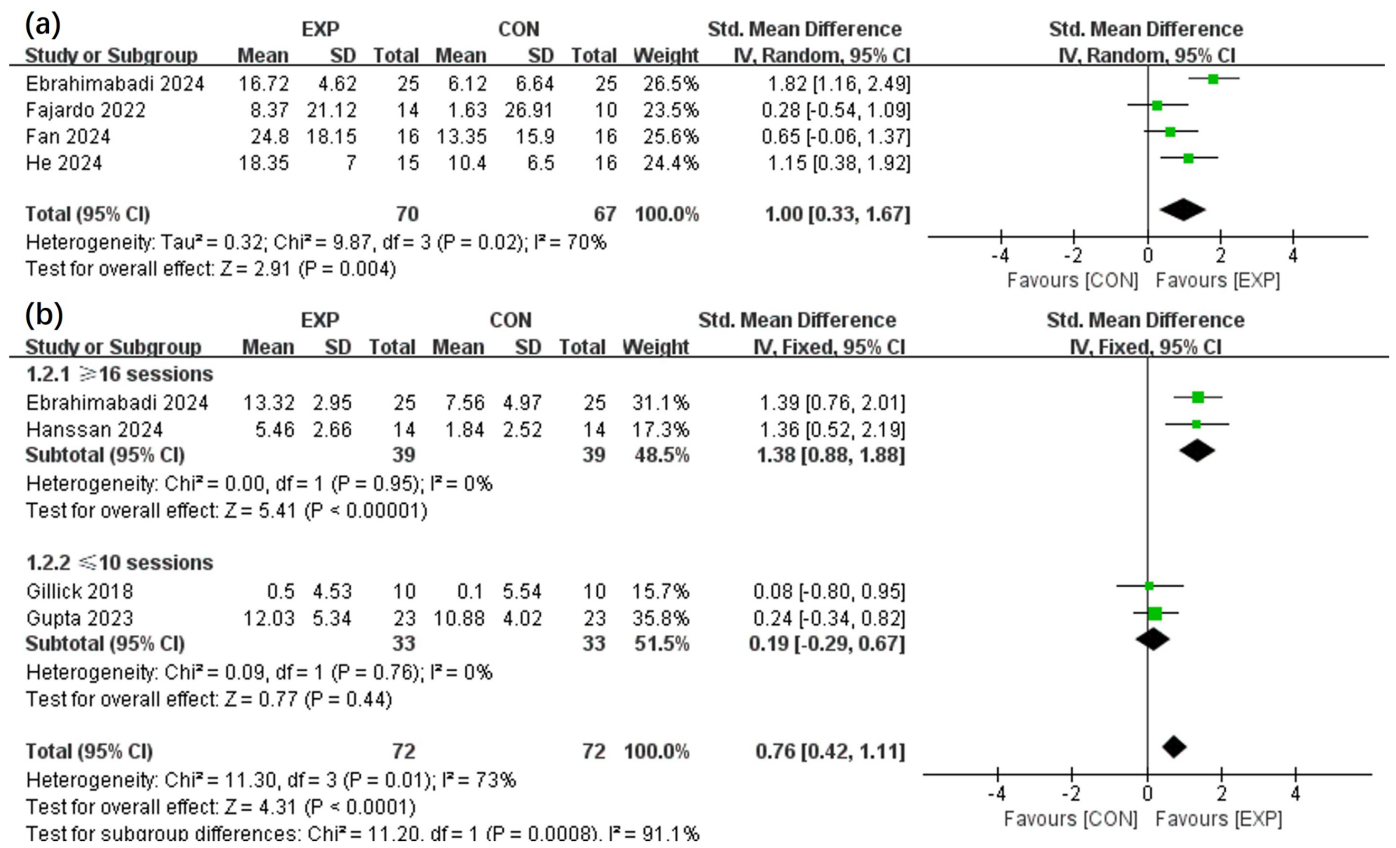
Forest plot illustrates the effects of EXP *versus* CON group on gross motor function (A) and grip strength (B). Ebrahimabadi, Ghaderian & Askary Kachoosangy, 2024; Salazar Fajardo et al., 2022; Fan et al., 2024; Gillick et al., 2018; Gupta et al., 2023; Hassan et al., 2024; He et al., 2024.

The effects of the intervention on GMF (Salazar Fajardo et al., 2022; Ebrahimabadi, Ghaderian & Askary Kachoosangy, 2024; Fan et al., 2024; He et al., 2024) and GS (Ebrahimabadi, Ghaderian & Askary Kachoosangy, 2024; Gupta et al., 2023; Gillick et al., 2018; Hassan et al., 2024) in children with CP within the EXP and CON groups were analysed across four selected articles. Figure 3 portrays that the EXP group exhibit a greater improvement in GMF relative to the CON group (SMD, 1.00 (0.33, 1.67), *p* < 0.05, I^2^ = 70%). The findings also implied that EXP significantly enhanced GS compared to CON (SMD, 0.76 (0.42, 1.11), *p* < 0.05, I^2^ = 73%). Subgroup analysis is based on clinical practice and data-driven results to address heterogeneity. Furthermore, subgroup analysis signified that interventions comprising above 16 sessions could notably improve GS (SMD, 1.38 (0.88, 1.88), *p* < 0.05, I^2^ = 0%), whereas those below 10 sessions did not demonstrate a significant effect (SMD, 0.19 (−0.29, 0.67), *p* = 0.44, I^2^ = 0%).

**Figure 4:**
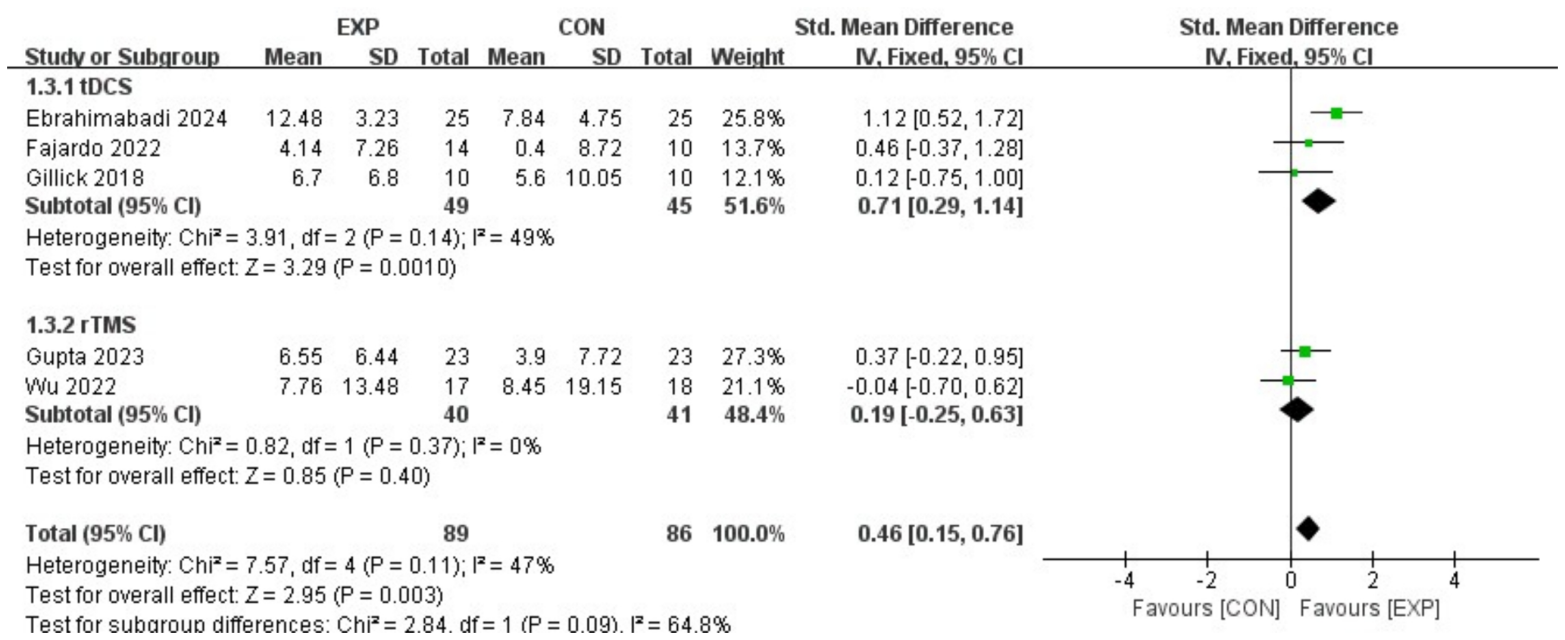
Forest plot illustrates the effects of EXP *versus* CON group on fine manual control. Ebrahimabadi, Ghaderian & Askary Kachoosangy, 2024; Salazar Fajardo et al., 2022; Gillick et al., 2018; Gupta et al., 2023; Wu et al., 2022.

Figure 4 illustrates a forest plot depicting the impact of brain stimulation and motor node on FMC in children with CP by incorporating data from five articles (Salazar Fajardo et al., 2022; Ebrahimabadi, Ghaderian & Askary Kachoosangy, 2024; Wu et al., 2022; Gupta et al., 2023; Gillick et al., 2018). Consequently, brain stimulation intervention could substantially enhance FMC (SMD, 0.46 (0.15, 0.76), *p* < 0.05, I^2^ = 47%). Subgroup analysis also presented that exercise-tDCS resulted in a significant improvement in FMC (SMD, 0.71 (0.29, 1.14), *p* < 0.05, I^2^ = 49%) compared to exercise-rTMS (SMD, 0.19 (−0.25, 0.63), *p* = 0.09, I^2^ = 47%).

### Analysis of publication bias

**Figure 5:**
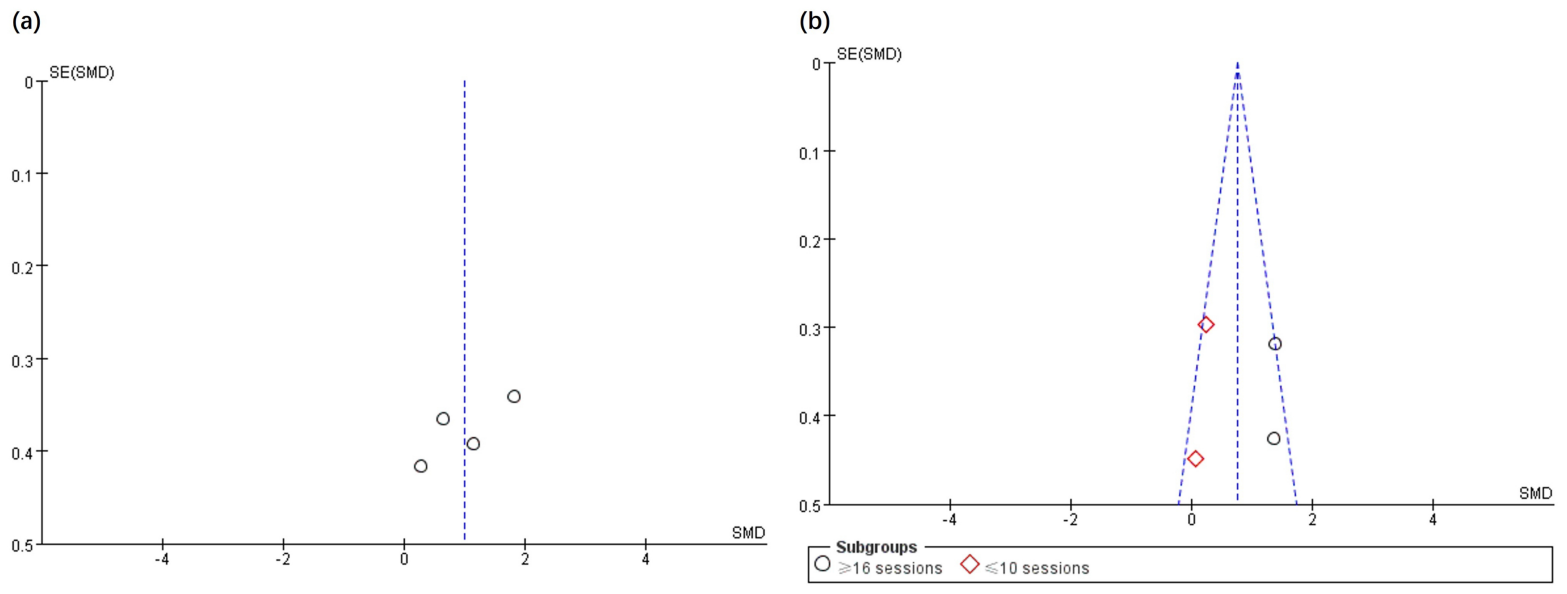
Funnel plot of publication bias for gross motor function (A) and grip strength (B) in the EXP *versus* CON group.

**Figure 6:**
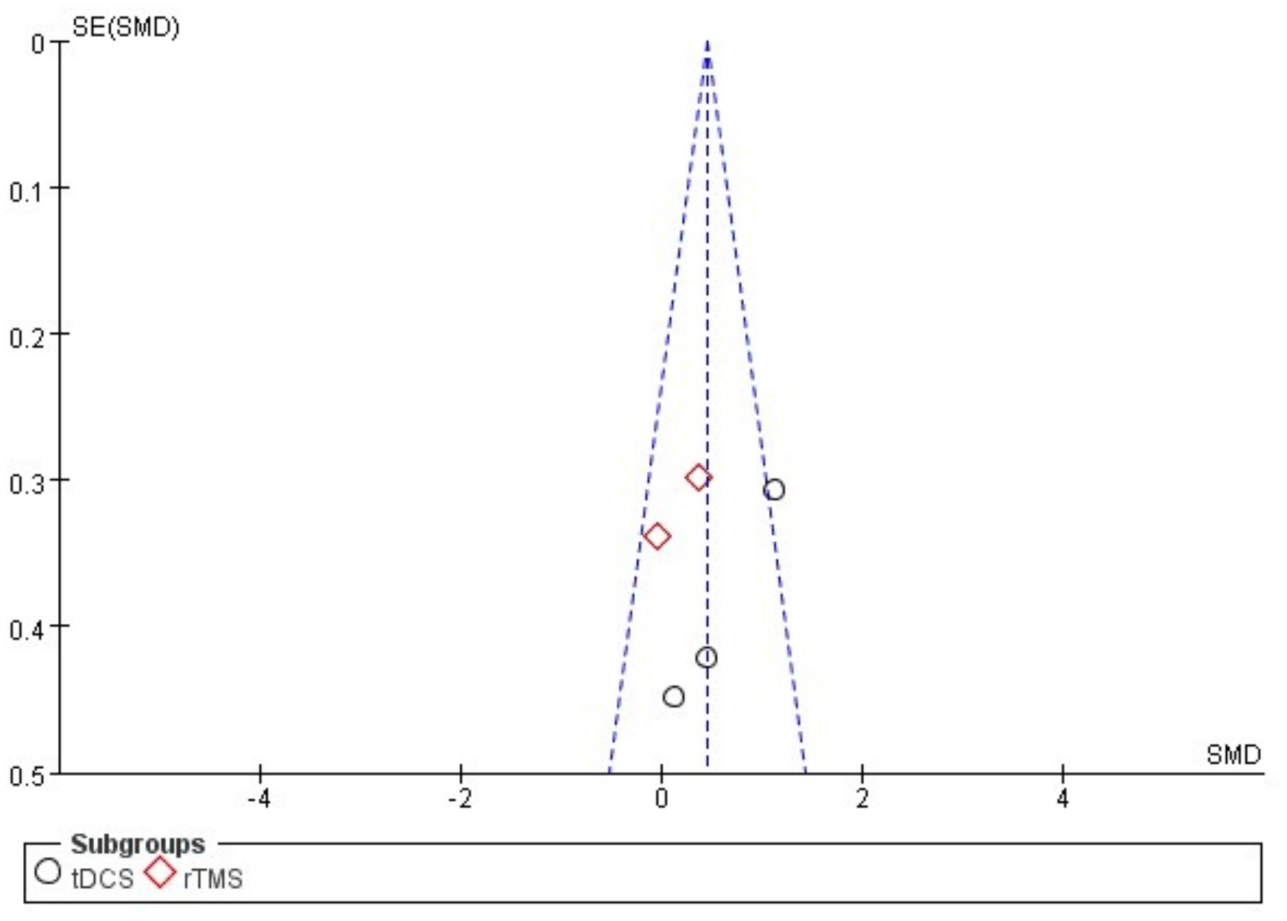
Funnel plot of publication bias for fine manual control in the EXP *versus* CON group.

The funnel plot was applied to assess the publication bias of the selected articles in the analysis. Specifically, the total number of selected articles approached the minimum threshold for using a funnel plot, indicating that publication bias could be assessed to a certain extent (Lu et al., 2020). Figs. 5 and 6 show no significant asymmetry, but the small number of studies limits the interpretation.

## Discussion

This review examined the impact of exercise-brain stimulation on hand function in children with CP. The primary indicators for assessing hand function were GMF, GS, and FMC. For results with high heterogeneity, the reasons for heterogeneity were evaluated by adjusting variables such as age, gender, intervention duration, intervention frequency, exercise time, type, intensity, and duration of brain stimulation. Despite exercise-brain stimulation substantially enhancing GMF, GS, and FMC, a framework involving fewer than 10 sessions did not demonstrate a significant impact on GS. Subgroup analysis also revealed a differential effect of tDCS and rTMS on FMC. Generally, brain plasticity serves as the theoretical foundation for physical therapy in children with CP, in which these children primarily exhibit hand dysfunction. The reduction in fine motor control and hand strength significantly impacts the daily activities and educational experiences in children with CP (Das & Ganesh, 2019). Hence, prompt and efficient rehabilitation treatment is pivotal to address this issue. Even though current physical therapy for children with CP primarily involves different movements and techniques, the functional areas of the cerebral cortex are often not directly stimulated. Therefore, external brain stimulation equipment is beneficial to address the limitations of traditional therapy. Examples of these advantages are promoting balance recovery, restoring cerebral hemisphere equilibrium, and enhancing motor function in children with CP (Tang et al., 2022).

This review demonstrated that exercise-brain stimulation could selectively enhance GMF, which aligned with the outcomes reported by previous studies. Salazar Fajardo et al. (2022) integrated neurodevelopmental treatment and tDCS, which was administered to children with CP over a period of five weeks (three times weekly). The study also employed the current intensity and duration of brain stimulation of 1 mA and 20 min, respectively. Consequently, the brain stimulation group exhibited a notable enhancement in the GMF score in comparison to the sham stimulation group. This phenomenon may be elucidated by the influence of anodal tDCS on the γ-aminobutyric acid (GABA) ergic system. Anodal tDCS may also result in a reduction of GABA levels in M1. Specifically, anode tDCS significantly reduced the GABA concentration in M1 region by inhibiting the activity of GABA synthase, which lasted for at least 60 minutes (Kim et al., 2014). Magnetic resonance spectroscopy study confirmed that the decrease of GABA concentration in M1 region after anode tDCS was significantly positively correlated with motor learning ability, that is, the greater the decrease of GABA, the better the performance of motor adaptation task and the stronger the retention of motor memory (Kim et al., 2014; Stagg, Bachtiar & Johansen-Berg, 2011). Buch et al. (2017) demonstrated that lower GABA levels within the primary motor cortex during anodal tDCS were associated with the extent of motor learning, as GABA reduction was essential for the occurrence of long-term potentiation (LTP).

The findings of this review presented the positive effects of exercise-tDCS on FMC, which agreed with several studies. Ebrahimabadi, Ghaderian & Askary Kachoosangy (2024) combined occupational therapy and tDCS, which was administered to children with CP over a period of four weeks (five times weekly). The tDCS current intensity was established at 1.5 mA, with the anode positioned in the O1 region of the left occipital lobe and the cathode in the O2 region of the right occipital lobe. Following the intervention, the real tDCS group produced a substantial improvement in FMC compared to the sham tDCS group. Typically, the interaction between exercise and tDCS in enhancing FMC can be attributed to the modulation of excitability within the primary motor cortex, which facilitates neural plasticity. Anodal tDCS can then enhance the neuronal discharge in the primary motor cortex, lower the action potential threshold, and improve the accuracy of motor commands. The study also noted that employing anodal tDCS-occupational therapy for children with unilateral CP notably increased the Fugl Meyer upper limb function score, with improvements observed to be twice that of the control group. Concurrently, a substantial enhancement was observed for hand grasping speed and coordination based on the box and block test. This effect pertained to the enhanced conduction efficiency of the cortical spinal pathway (immediately and 24 h post-stimulation). Furthermore, the tDCS could facilitate synaptic remodelling through the activation of the brain-derived neurotrophic factor/tropomyosin receptor kinase B pathway. In addition, exercise and tDCS seem to have a cumulative effect, and the simultaneous application can bring better benefits to FMC. tDCS creates a neural plasticity window for exercise training by regulating cortical excitability. The synergy of TDCS and sports training is based on the theory of “neural plasticity window” (Marchiotto, Cambiaghi & Buffelli, 2025; Steinberg, Pixa & Fregni, 2018). Anode tDCS creates a highly plastic state in the motor cortex by reducing GABA inhibition, enhancing BDNF expression and promoting LTP (Marchiotto, Cambiaghi & Buffelli, 2025). Subsequent exercise training, as an intensive stimulus, repeatedly activates specific neural pathways during this window period, significantly enhancing the training effect. Studies have shown that this synergistic effect magnifies the effect of neural plasticity by 2–3 times, far more than using tDCS or exercise training alone (Steinberg, Pixa & Fregni, 2018). Anodic stimulation depolarizes M1 neurons and triggers LTP to improve synaptic transmission efficiency; Cathodic stimulation can induce long-term depression through GABA inhibition and correct the hemispheric imbalance. Exercise training uses this window to repeatedly strengthen action memory, and the two cooperate to optimize the accuracy of neuromuscular regulation and further improve FMC (Reis et al., 2009; Madhavan, Weber & Stinear, 2011).

A study (Liang et al., 2022) documented that tDCS-exercise training could enhance the maturation of newborn neurons in the hippocampus, which was a process closely associated with the release of brain-derived neurotrophic factor. This review also indicated that an optimal level of exercise and brain stimulation positively influenced GS (Zhang et al., 2022). Meanwhile, a significant relationship has been suggested between GS and hand function for children with CP (Jesunathadas et al., 2010). Enhanced neuroplasticity can then optimise motor unit recruitment and coordination among hand muscle groups. The regulation of cerebello cortical circuit by tDCS can also increase temporal accuracy of force control. Conversely, stimulation of the dorsolateral prefrontal cortex (DLPFC) improves executive function and motor planning, optimising GS stability and accuracy (Gbadeyan et al., 2016; Schmitter & Straube, 2022). Overall, the combination of exercise and tDCS, especially in the program with sufficient intervention duration, seems to be a promising non drug rehabilitation method for children with cerebral palsy.

This review presented multiple limitations. Initially, the sample size was limited (comprising only eight articles), and the units of measurement were inconsistent. Notably, the main analyses exhibited high heterogeneity, which limits the strength of the overall conclusions. Consequently, only SMDs were applicable for comparison. Another constraint was that this review only included children with CP, limiting the applicability of the results to other populations. Although the intervention of less than 10 sessions did not show a statistically significant impact on GS index, it could not exclude the clinically meaningful effect. The intervention protocol in tDCS for this review also exhibited inconsistencies between low current and high current protocols, restricting cross-sectional comparisons among articles. Additionally, the number of studies in each subgroup was small, which limits the statistical power and generalizability of the subgroup findings. In addition, the mechanism through which exercise-brain stimulation enhanced hand function in children with CP remained unclear. Therefore, the impact of exercise-brain stimulation across various populations warrants further investigation in future studies, and the underlying mechanisms should be analysed in greater detail.

## Conclusion

This review demonstrated that exercise-brain stimulation could significantly enhance hand function in children with CP. Specifically, more than 16 sessions has greater benefits for GS, and tDCS may confer benefits for FMC. Therefore, a rehabilitation protocol combining exercise with tDCS, delivered in more than 16 sessions, represents a viable non-pharmacological strategy to enhance hand function in children with CP.

##  Supplemental Information

10.7717/peerj.20670/supp-1 Supplemental Information 1 PRISMA checklist

10.7717/peerj.20670/supp-2 Supplemental Information 2 Search strategy

10.7717/peerj.20670/supp-3 Supplemental Information 3 Grade evidence profile

10.7717/peerj.20670/supp-4 Supplemental Information 4 Target audience information

10.7717/peerj.20670/supp-5 Supplemental Information 5 Data

## Additional Information and Declarations

### Data Availability

The following information was supplied regarding data availability: This is a systematic review/meta-analysis.

## References

[ref-1] Brunoni AR, Ekhtiari H, Antal A, Auvichayapat P, Baeken C, Benseñor IM, Bikson M, Boggio P, Borroni B, Brighina F, Brunelin J, Carvalho S, Caumo W, Ciechanski P, Charvet L, Clark VP, Cohen Kadosh R, Cotelli M, Datta A, Deng Z-D, De Raedt R, De Ridder D, Fitzgerald PB, Floel A, Frohlich F, George MS, Ghobadi-Azbari P, Goerigk S, Hamilton RH, Jaberzadeh SJ, Hoy K, Kidgell DJ, Zonoozi AK, Kirton A, Laureys S, Lavidor M, Lee K, Leite J, Lisanby SH, Loo C, Martin DM, Miniussi C, Mondino M, Monte-Silva K, Morales-Quezada L, Nitsche MA, Okano AH, Oliveira CS, Onarheim B, Pacheco-Barrios K, Padberg F, Nakamura-Palacios EM, Palm U, Paulus W, Plewnia C, Priori A, Rajji TK, Razza LB, Rehn EM, Ruffini G, Schellhorn K, Zare-Bidoky M, Simis M, Skorupinski P, Suen P, Thibaut A, Valiengo LCL, Vanderhasselt M-A, Vanneste S, Venkatasubramanian G, Violante IR, Wexler A, Woods AJ, Fregni F (2022). Digitalized transcranial electrical stimulation: a consensus statement. Clinical Neurophysiology.

[ref-2] Buch ER, Santarnecchi E, Antal A, Born J, Celnik PA, Classen J, Gerloff C, Hallett M, Hummel FC, Nitsche MA, Pascual-Leone A, Paulus WJ, Reis J, Robertson EM, Rothwell JC, Sandrini M, Schambra HM, Wassermann EM, Ziemann U, Cohen LG (2017). Effects of tDCS on motor learning and memory formation: a consensus and critical position paper. Clinical Neurophysiology.

[ref-3] Burgess A, Boyd RN, Chatfield MD, Ziviani J, Wotherspoon J, Sakzewski L (2021). Hand function and self-care in children with cerebral palsy. Developmental Medicine and Child Neurology.

[ref-4] Chiara R, Kotzalidis GD, Ferracuti S, Sani G, Girardi P, Del Casale A (2019). Brain stimulation in obsessive-compulsive disorder (OCD): a systematic review. Current Neuropharmacology.

[ref-5] Das SP, Ganesh GS (2019). Evidence-based approach to physical therapy in cerebral palsy. Indian Journal of Orthopaedics.

[ref-6] Ebrahimabadi Z, Ghaderian B, Askary Kachoosangy R (2024). Transcranial direct current stimulation combined with occupational therapy improves upper limb function in children with unilateral cerebral palsy: a randomized controlled trial. Iranian Journal of Pediatrics.

[ref-7] Fan T, Wei H, Dai J, You G, Lu Z (2024). Repeated transcranial magnetic stimulation combined with action observation training in children with spastic cerebral palsy. Journal of Visualized Experiments.

[ref-8] Figueiredo PRP, Mancini MC, Feitosa AM, Teixeira CMMF, Guerzoni VPD, Elvrum A-KG, Ferre CL, Gordon AM, Brandão MB (2020). Hand-arm bimanual intensive therapy and daily functioning of children with bilateral cerebral palsy: a randomized controlled trial. Developmental Medicine and Child Neurology.

[ref-9] Gbadeyan O, McMahon K, Steinhauser M, Meinzer M (2016). Stimulation of dorsolateral prefrontal cortex enhances adaptive cognitive control: a high-definition transcranial direct current stimulation study. Journal of Neuroscience.

[ref-10] Gillick B, Rich T, Nemanich S, Chen C-Y, Menk J, Mueller B, Chen M, Ward M, Meekins G, Feyma T, Krach L, Rudser K (2018). Transcranial direct current stimulation and constraint-induced therapy in cerebral palsy: a randomized, blinded, sham-controlled clinical trial. European Journal of Paediatric Neurology.

[ref-11] Gupta J, Gulati S, Singh UP, Kumar A, Jauhari P, Chakrabarty B, Pandey RM, Bhatia R, Jain S, Srivastava A (2023). Brain stimulation and constraint induced movement therapy in children with unilateral cerebral palsy: a randomized controlled trial. Neurorehabilitation and Neural Repair.

[ref-12] Hanna SE, Law MC, Rosenbaum PL, King GA, Walter SD, Pollock N, Russell DJ (2003). Development of hand function among children with cerebral palsy: growth curve analysis for ages 16 to 70 months. Developmental Medicine and Child Neurology.

[ref-13] Hassan Z, Hadian M-R, Hussain SA, Shadmehr A, Talebian S, Bagheri H, Mir SM, Arslan SA (2024). Comparison of the conjunct effects of electrical stimulation and whole-body vibration therapy with transcranial direct current stimulation and whole-body vibration therapy on balance and function in children with spastic cerebral palsy. Cureus.

[ref-14] He Y, Zhang Q, Ma T-T, Liang Y-H, Guo R-R, Li X-S, Liu Q-J, Feng T-Y (2024). Effect of repetitive transcranial magnetic stimulation-assisted training on lower limb motor function in children with hemiplegic cerebral palsy. BMC Pediatrics.

[ref-15] Higgins JPT, Thomas J, Chandler J, Cumpston M, Li T, Page MJ, Welch VA (2019). Cochrane handbook for systematic reviews of interventions.

[ref-16] Hilderley AJ, Wright FV, Taylor MJ, Chen JL, Fehlings D (2023). Functional neuroplasticity and motor skill change following gross motor interventions for children with diplegic cerebral palsy. Neurorehabilitation and Neural Repair.

[ref-17] Ilieva E, Ilieva A (2020). What is the effect of constraint-induced movement therapy on children with unilateral cerebral palsy? A cochrane review summary with commentary. Developmental Medicine and Child Neurology.

[ref-18] Jesunathadas M, Marmon AR, Gibb JM, Enoka RM (2010). Recruitment and derecruitment characteristics of motor units in a hand muscle of young and old adults. Journal of Applied Physiology.

[ref-19] Kim S, Stephenson MC, Morris PG, Jackson SR (2014). tDCS-induced alterations in GABA concentration within primary motor cortex predict motor learning and motor memory: a 7 T magnetic resonance spectroscopy study. NeuroImage.

[ref-20] Li S, Shaharudin S, Cirer-Sastre R, Li F, Abdul Manaf F, Mohd Shukri MF (2023). Effects of high-intensity interval exercise on cardiac troponin elevation when comparing with moderate-intensity continuous exercise: a systematic review and meta-analysis. PeerJ.

[ref-21] Liang X, Tang J, Qi Y-Q, Luo Y-M, Yang C-M, Dou X-Y, Jiang L, Xiao Q, Zhang L, Chao F-L, Zhou C-N, Tang Y (2022). Exercise more efficiently regulates the maturation of newborn neurons and synaptic plasticity than fluoxetine in a CUS-induced depression mouse model. Experimental Neurology.

[ref-22] Lu Y, Wang W, Ding X, Shi X (2020). Association between the promoter region of serotonin transporter polymorphisms and recurrent aphthous stomatitis: a meta-analysis. Archives of Oral Biology.

[ref-23] Madhavan S, Weber KA, Stinear JW (2011). Non-invasive brain stimulation enhances fine motor control of the hemiparetic ankle: implications for rehabilitation. Experimental Brain Research.

[ref-24] Marchiotto F, Cambiaghi M, Buffelli M (2025). Physical activity and anodal-transcranial direct current stimulation: a synergistic approach to boost motor cortex plasticity. Brain Communications.

[ref-25] Ostensjø S, Carlberg EB, Vøllestad NK (2004). Motor impairments in young children with cerebral palsy: relationship to gross motor function and everyday activities. Developmental Medicine and Child Neurology.

[ref-26] Reis J, Schambra HM, Cohen LG, Buch ER, Fritsch B, Zarahn E, Celnik PA, Krakauer JW (2009). Noninvasive cortical stimulation enhances motor skill acquisition over multiple days through an effect on consolidation. Proceedings of the National Academy of Sciences of the United States of America.

[ref-27] Salazar Fajardo JC, Kim R, Gao C, Hong J, Yang J, Wang D, Yoon B (2022). The effects of tDCS with NDT on the improvement of motor development in cerebral palsy. Journal of Motor Behavior.

[ref-28] Schmitter CV, Straube B (2022). The impact of cerebellar transcranial direct current stimulation (tDCS) on sensorimotor and inter-sensory temporal recalibration. Frontiers in Human Neuroscience.

[ref-29] Stagg CJ, Bachtiar V, Johansen-Berg H (2011). The role of GABA in human motor learning. Current Biology.

[ref-30] Steinberg F, Pixa NH, Fregni F (2018). A review of acute aerobic exercise and transcranial direct current stimulation effects on cognitive functions and their potential synergies. Frontiers in Human Neuroscience.

[ref-31] Tang L, Wu Y, Ma J, Lu Y, Wang L, Shan C (2022). Application of tDCS in children with cerebral palsy: a mini review. Frontiers in Pediatrics.

[ref-32] Wu Q, Peng T, Liu L, Zeng P, Xu Y, Yang X, Zhao Y, Fu C, Huang S, Huang Y, Zhou H, Liu Y, Tang H, He L, Xu K (2022). The effect of constraint-induced movement therapy combined with repetitive transcranial magnetic stimulation on hand function in preschool children with unilateral cerebral palsy: a randomized controlled preliminary study. Frontiers in Behavioral Neuroscience.

[ref-33] Zhang Y, Li R, Miao X, Cheng LJ, Lau Y (2022). Virtual motor training to improve the activities of daily living, hand grip, and gross motor function among children with cerebral palsy: meta-regression analysis. Gait & Posture.

[ref-34] Zhong M, Zhang Y, Luo J, Chen T, Zhang J, Peng T, Han M, Le W, Peng T, Xu K (2025). Safety and effectiveness of non-invasive brain stimulation on mobility and balance function in children with cerebral palsy: a systematic review and meta-analysis. Journal of NeuroEngineering and Rehabilitation.

