# Supplementary material for: Effects of exercise combined with brain stimulation on hand function in children with cerebral palsy: a meta-analysis of randomized controlled trials
Source: PeerJ. 2026 Jan 29;14:e20670. doi: 10.7717/peerj.20670 (PMC12861132; doi:10.7717/peerj.20670)
Supplement: Supplemental Information 2 [file peerj-14-20670-s002.docx]

**Appendix A**

Search strategy

| Databases | Search strategy | Result  (Approximately) |
| --- | --- | --- |
| Scopus | #1: Title-Abs-Key (Cerebral palsy)  #2: Title-Abs-Key (Stimulation)  #3: Title-Abs-Key (Week*)  #5: #1 and #2 and #3  Limiters - Published Date: 20150101-20250629 | 19,763  319,746  866,817  207 |
| Pubmed | #1: [Title/Abstract] Cerebral palsy  #2: [Title/Abstract] Stimulation  #3: [Title/Abstract] Week*  #4: #1 and #2 and #3  Filters: Publication date from 2015/01/01 to 2025/06/29 | 14,184  194,242  693,642  147 |
| Web of Science | #1: TOPIC: (Cerebral palsy)  #2: TOPIC: (Stimulation)  #3: TOPIC: (Week*)  #4: #1 and #2 and #3  Refined by: PUBLICATION YEARS: (20250629-20150101)  Indexes=SCI-EXPANDED, SSCI, CCR-EXPANDED, | 17,294  245,275  659,526  197 |
| EBSCO | #1: Abstract: (Cerebral palsy)  #2: Abstract: (Stimulation)  #3: Abstract: (Week*)  #4: #1 and #2 and #3  Year: 20150101-20250629 | 16,317  194,295  739,435  139 |
