# Supplementary material for: Effects of exercise combined with brain stimulation on hand function in children with cerebral palsy: a meta-analysis of randomized controlled trials
Source: PeerJ. 2026 Jan 29;14:e20670. doi: 10.7717/peerj.20670 (PMC12861132; doi:10.7717/peerj.20670)
Supplement: Supplemental Information 3 [file peerj-14-20670-s003.docx]

**Appendix B Grade evidence profile**

| **Certainty assessment** | | | | | | | **№ of patients** | | **Effect** | | **Certainty** | **Importance** |
| --- | --- | --- | --- | --- | --- | --- | --- | --- | --- | --- | --- | --- |
| **№ of studies** | **Study design** | **Risk of bias** | **Inconsistency** | **Indirectness** | **Imprecision** | **Other considerations** | **Theory-based interventions** | **Routine care** | **Relative (95% CI)** | **Absolute (95% CI)** |  |  |
| Gross motor function | | | | | | | | | | | | |
| 4 | randomised trials | not serious | not serious | not serious | serious^c^ | none | 70 | 67 | - | SMD **1.00 higher** (0.33 higher to 1.67 higher) | ⨁⨁⨁◯ Moderate |  |
| Grip strength | | | | | | | | | | | | |
| 4 | randomised trials | serious^a^ | not serious | not serious | serious^c^ | none | 72 | 72 | - | SMD **0.76 higher** (0.42 higher to 1.11 higher) | ⨁⨁◯◯  Low |  |
| Fine manual control | | | | | | | | | | | | |
| 5 | randomised trials | not serious | not serious | not serious | serious^c^ | none | 89 | 86 | - | SMD **0.46 lower** (0.15 higher to 0.76 higher) | ⨁⨁⨁◯  Moderate |  |

**CI:** confidence interval; **SMD:** standardised mean difference

a. Downgrade one level for most of the study have unclear or high risk of bias

b. Downgrade one level for the inconsistency due to considerable heterogeneity (I^2^ > 50%)

c. Downgrade one level for the imprecision due to the total sample size does not meet the rules of thumb (at least 400 participants)
