# Supplementary material for: Effects of exercise combined with brain stimulation on hand function in children with cerebral palsy: a meta-analysis of randomized controlled trials
Source: PeerJ. 2026 Jan 29;14:e20670. doi: 10.7717/peerj.20670 (PMC12861132; doi:10.7717/peerj.20670)
Supplement: Supplemental Information 4 [file peerj-14-20670-s004.docx]

Exercise combined with brain stimulation interventions for motor function improvement are key focuses in cerebral palsy (CP) rehabilitation. Children with CP often face challenges in daily activity independence. This study/table summarizes core characteristics of related randomized trials, providing targeted references for pediatric rehabilitation physicians, physical therapists, occupational therapists who need to design evidence-based combined intervention programs. It also offers reliable intervention direction for parents/guardians of children with CP concerned about motor function improvement, as well as basic data support for researchers engaged in CP neurorehabilitation or brain stimulation technology research.
